# Atherosclerotic plaque evolution predicts cerebral ischemic events in patients with intracranial atherosclerosis: a multicentre longitudinal study using high-resolution MRI

**DOI:** 10.1007/s00330-024-11248-8

**Published:** 2024-12-19

**Authors:** Weihe Yao, Hongbing Chen, Kangmo Huang, Wenjia Peng, Xuefeng Zhang, Dahong Yang, Zhongzhao Teng, Jinhua Shen, Jialuo Yang, Xiaoqing Cheng, Yunfei Han, Wusheng Zhu, Junjun Wang, Juan Du, Xinfeng Liu

**Affiliations:** 1 Department of Neurology, Jinling Hospital, Affiliated Hospital of Medical School, Nanjing University, Nanjing, China; 2 Department of Neurology, The First Affiliated Hospital, Sun Yat-sen University, Guangzhou, China; 3 Department of Radiology, Changhai Hospital, Shanghai, China; 4 Department of Radiology, University of Cambridge, Cambridge, UK; 5 Nanjing Jingsan Medical Science and Technology Ltd., Jiangsu, China; 6 Department of Medical Imaging, Jinling Hospital, Affiliated Hospital of Medical School, Nanjing University, Nanjing, China; 7 State Key Laboratory of Pharmaceutical Biotechnology, Department of Clinical Laboratory, Jinling Hospital, Affiliated Hospital of Medical School, Nanjing University, Nanjing, China

**Keywords:** Intracranial atherosclerosis, Magnetic resonance imaging, Stroke, Recurrence

## Abstract

**Background:**

Intracranial atherosclerosis (ICAS) is the leading cause of ischemic stroke in Asians and the recurrent rate remains high despite the optimal medical treatment. This study aimed to confirm that follow-up high-resolution magnetic resonance imaging (hrMRI) provided essential values in predicting subsequent cerebral ischemic events in patients with ICAS.

**Methods:**

Patients with moderate to severe stenosis in the middle cerebral artery (MCA) defined by magnetic resonance (MRA) or computed tomography angiography (CTA) were recruited from three centers retrospectively. Detailed plaque composition was analyzed on baseline and follow-up hrMRI. Multivariate Cox proportional hazards regression analysis was used to identify the key risk factors for predicting subsequent ischemic events.

**Results:**

Among 152 patients, a total of 86 patients with MCA atherosclerotic stenosis underwent follow-up hrMRI exams and ipsilateral cerebral ischemic events occurred in 12 patients during a 1-year follow-up. Analyses showed the predictors of ischemic events were age (adjusted Hazard ratio (HR) = 0.942; 95% Confidence Interval (CI), [0.903, 0.983]; *p* = 0.006), progression of plaque burden (HR = 3.818; 95% CI [1.117, 13.051]; *p* = 0.033), vessel expansion (HR = 5.173; 95% CI [1.077, 24.838]; *p* = 0.040) and enhancement ratio progression (HR = 6.144; 95% CI [1.480, 25.511]; *p* = 0.012). The combined model achieved a concordance index of 0.804 (95% CI [0.658, 0.950]).

**Conclusion:**

Longitudinal hrMRI evaluation improved the accuracy in identifying higher-risk patients with intracranial atherosclerosis.

**Key Points:**

***Question*** *Can longitude high-resolution magnetic resonance imaging (hrMRI) help clinicians observe intracranial plaque evolution?*

***Findings*** *Compared with the baseline exam, intracranial plaque evolution distinguished by follow-up hrMRI exam showed a higher accuracy in predicting subsequent ischemic events.*

***Clinical relevance*** *Longitudinal high-resolution magnetic resonance vessel wall imaging enables dynamic observation and evaluation of the changes in plaque characteristics among intracranial atherosclerosis patients. Atherosclerotic plaque evolution revealed by repeated exams can strengthen the risk stratification of patients with intracranial atherosclerosis.*

## Introduction

Intracranial atherosclerosis stenosis (ICAS) is one of the primary etiologies of ischemic stroke worldwide and is the leading cause in the East Asian population [1–4]. ICAS mainly occurs in the middle cerebral artery (MCA). Despite optimal medical treatment, symptomatic patients with ICAS still have a high risk of recurrent ischemic events with ~15% in the first year [5]. The suboptimal treatment outcome could be attributed to the current constraints in prognostic evaluation techniques.

In current clinical practice, luminal stenosis is the main determinant for risk assessment. However, increasing evidence has demonstrated that detailed lesion morphological and compositional features are more relevant to patient clinical presentation and subsequent ischemic events in the coronary [6], carotid [7–9], and intracranial territories [10, 11]. High-resolution magnetic resonance vessel wall imaging (hrMRI) can depict this morphological and compositional information [4, 10, 12]. Several studies have investigated the correlation between plaque features and the recurrence of ischemic stroke through the utilization of hrMRI. High-risk features include plaque enhancement, steepness, and intraplaque hemorrhage, although inconsistent findings exist across different studies [13, 14]. Nevertheless, hrMRI has shown great potential for precisely personalized medical therapy [15].

Atherosclerosis is a dynamic complex structure [6, 16] influenced by systematic risk factors, e.g., cholesterol and glucose levels, local hemodynamic environment, lesion inflammation, etc. Moreover, different medications and treatment compliance may lead to various responses in plaque features [17, 18]. Recent studies have demonstrated that the progression or regression of coronary or carotid plaques may have a stronger association with recurrent ischemic events [19]. Despite these dynamic observations, most of the previous reports are cross-sectional single-center studies with small sample sizes, and there were few longitudinal hrMRI studies on the evolution of intracranial atherosclerosis and the associated clinical significance [20–22]. This multicentre longitudinal follow-up hrMRI study was, therefore, designed to investigate the clinical relevance of the intracranial plaque evolution in predicting subsequent cerebral ischemic events in patients with ICAS.

## Materials and methods

### Patient enrollment and follow-up

Between January 2012 and August 2020, patients who underwent repeated hrMRI exams were retrospectively enrolled from three hospitals in China, Changhai Hospital, Shanghai (January 2012 to August 2013); Jinling Hospital, Nanjing (March 2014 to August 2020); and Zhongshan Hospital, Guangzhou (March 2014 to August 2020). This study was approved by the Ethics Committee of each participating center and written informed consent was waived owing to the retrospective nature of the study.

The inclusion criteria were: (1) Age ≥ 18 years old; (2) confirmed MCA stenosis (50–99%) by magnetic resonance (MRA) or computed tomography angiography (CTA); (3) with at least one cardiovascular risk factor, including hypertension, diabetes, hyperlipidemia, obesity, smoking, and coronary artery disease; and (4) underwent repeated hrMRI exam within 1 year. The exclusion criteria were: (1) confirmed non-atherosclerotic MCA stenosis, including moya-moya disease, vasculitis, arterial dissection, or other disease; (2) coexisting ipsilateral moderate to severe carotid stenosis (> 50%). (3) underwent intravascular treatment (balloon or stenting) previously or during follow-up; (4) poor image quality; and (5) loss of follow-up.

The patients’ baseline clinical data were collected. All patients were interviewed at the 3rd, 6th, and 12th months after baseline hrMRI exam either through clinical visits or telephone interviews. The primary endpoints were ipsilateral ischemic events including ischemic stroke or TIA, which were assessed by neurologists in each center.

### High-resolution magnetic resonance vessel wall imaging (hrMRI)

All patients from three centers were scanned by a 3.0-T MRI scanner (GE or SIEMENS MR system) with the multi-channel head coil. The following sequences were contained in the exam protocol: three-dimension time-of-flight angiography (3D TOF MRA), sagittal two-dimension high-resolution black blood T_1_- (T1WI), T_2_-weighted (T2WI) and contrast-enhanced T_1_-weighted (CE-T1WI) sequences perpendicular to the longitudinal orientation of MCA M1 segment, and standard brain MRI including diffusion-weighted imaging (DWI). Specific scanning parameters of each center can be found in the Supplementary material.

### Image analysis

Imaging quality was ranked with a 4-point scheme [23], patients with poor image quality (< 3) were excluded from the analysis. Two experienced MR readers blinded to patient clinical information (W.Y. and K.H., with 5 and 4 years of experience in neurology and neuroradiology, respectively) analyzed hrMRI images. Disagreement, if present, was solved by a discussion with a third senior radiologist (J.D., with 16 years of experience in neuroradiology).

The degree of stenosis was measured on the maximum intensity projection of 3D TOF MRA following Warfarin-Aspirin Symptomatic Intracranial Disease (WASID) criteria [24], stenosis rate (%) = (1-diameter of stenotic artery/diameter of proximal normal artery) × 100%. The lumen and vessel outer wall boundaries at the most stenotic site were identified as well as boundaries of the nearby disease-free reference section, and the corresponding areas were calculated, including minimum luminal area (MLA), vessel area (VA), and reference area (RA). At the most stenotic site, the maximum wall thickness (WT_max_) and minimum wall thickness (WT_min_) were also calculated. The following hrMRI parameters were further calculated, including: Plaque burden (PB) = (VA_MLA_-LA_MLA_)/(VA_MLA_); Remodeling index = (VA_MLA_)/(VA_RA_); Eccentricity index = (WT_max_-WT_min_)/WT_max_ [25, 26]. If the eccentricity index > 0.5, the lesion was defined as an eccentric plaque [27].

In the black blood T1WI and T1-CEWI sequence, the signal intensity of the lesion and reference site were also measured. Intraplaque hemorrhage (IPH) was defined as hyperintensity in T1WI (lesion greatest signal/nearby brain parenchyma ≥ 1.5) [28]. The plaque enhancement ratio was calculated as the ratio between the lesion’s greatest enhancement signal with reference to the nearby brain parenchyma on the CE-T1WI sequence [20]. The plaque enhancement was defined as an enhancement ratio over 1.5. According to the enhancement ratio, the enhancement degree was graded as follows: 0 = no enhancement (enhancement ratio < 1.5), 1 = mild enhancement (1.5 ≤ enhancement ratio < 2.0), and 2 = obvious enhancement (enhancement ratio ≥ 2.0). All image analyses were performed on MR-VascularView (Jingsan Medical Science and Technology, Ltd.).

The relative difference between the aforementioned parameters at baseline and follow-up was calculated as, [(value at follow-up-value at baseline)/value at baseline] × 100%. Progression of plaque burden was defined as an increase from baseline to follow-up over 5% [29]. According to the change percentage of VA_MLA_, lesions were divided into three groups, vessel expansion (percentage ≥ 5%), vessel unchanged (−5% < percentage < 5%), and vessel shrinkage (percentage ≤ −5%). According to the change percentage of enhancement ratio, lesions were divided as, enhancement progression (percentage ≥ 5%), enhancement unchanged (−5% < percentage < 5%), and enhancement regression (percentage ≤ −5%).

### Statistical analysis

Statistical analyses were performed using SPSS 26.0.0 (IBM), MedCalc® Statistical Software version 20.111 (MedCalc Software Ltd.). Continuous variables were reported as mean ± standard deviation (SD) or median [interquartile range] according to the distribution assessed using the Kolmogorov-Smirnov test. In the univariate test, the Student’s *t*-test or Mann–Whitney U chi-square test was used for continuous variables, and the χ^2^ test or Fisher exact test was used for categorical variables where appropriate. Paired Student’s *t*-test was used for paired samples. Intraclass correlation coefficient (ICC) and Cohen’s kappa test were used to evaluate the intra- and inter-observer agreement for continuous and categorical variables. X-tile software (version 3.6.1) was used to determine the optimal cutoff value for continuous parameters. Kaplan–Meier curves were then plotted and analyzed by the Log-rank test. Variables with *p* < 0.1 in the univariate Cox regression were further incorporated into the multivariate Cox regression. Results were expressed as hazard ratio (HR) with a 95% confidence interval (95% CI). The concordance index (C-index) was used to evaluate the accuracy of the Cox model. All tests were two-sided, and *p* < 0.05 was assumed to be statistically significant.

## Results

**Fig. 1:**
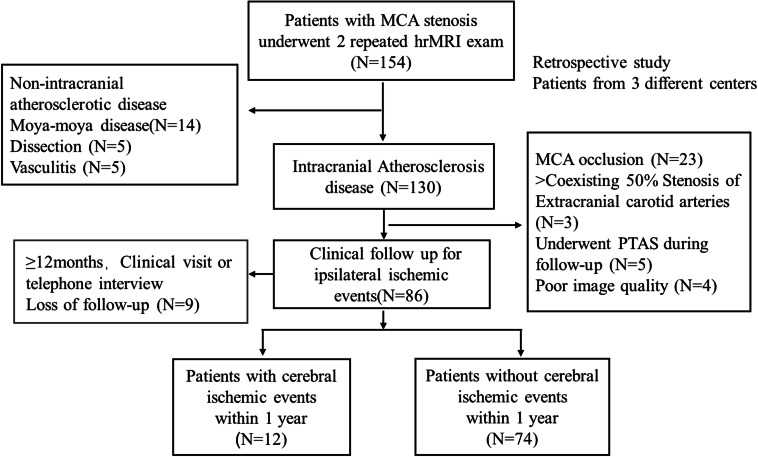
Flowchart of the study population selection and grouping

**Fig. 2:**
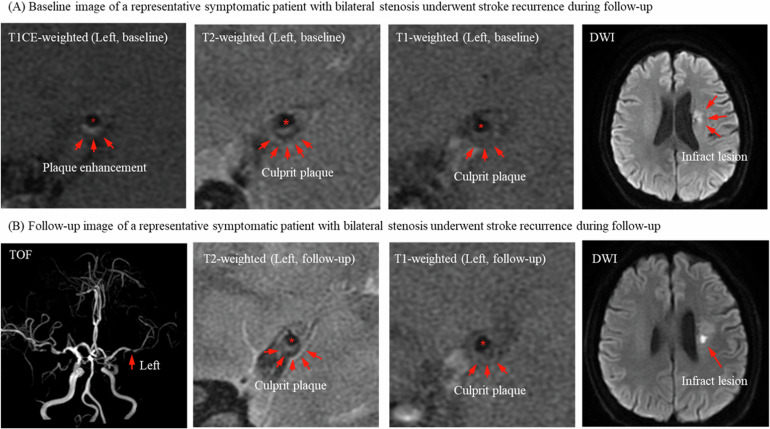
Baseline and follow-up high-resolution magnetic resonance imaging (hrMRI) of a patient with recurrent ischemic stroke on the same territory. **A** 60-year-old male patient with ischemic stroke underwent hrMRI examination upon admission. TOF-MRA found bilateral middle cerebral artery (MCA) stenosis and left MCA was regarded as the culprit lesion. The images demonstrate the appearance of culprit plaque in the left hemisphere on black blood 2-dimensional T2, and T1 sequences (red arrows). The diffusion-weighted imaging (DWI) sequence shows cerebral infarction within the left MCA supply area (red arrow). **B** The patient experienced symptomatic recurrence and was readmitted 2 months after discharge for further hrVWI examination, which confirmed recurrent ischemic stroke within the same territory. TOF-MRA and Black blood T2-weighted imaging and T1 sequences show the progression of stenosis and plaque burden compared to baseline images (red arrow), while the DWI sequence demonstrates a new ischemic lesion (red arrow). *Lumen

A total of 154 participants from three centers were recruited and 68 patients were excluded due to: (1) non-intracranial atherosclerotic disease (*n* = 24); (2) MCA occlusion or coexisting extracranial carotid arteries (*n* = 26); (3) underwent PTAS during follow-up (*n* = 5); (4) loss of follow-up (*n* = 9); and (5) poor image quality (*n* = 4). Finally, a total of 86 patients with MCA moderate to severe stenosis underwent repeated hrMRI exams were included (Fig. 1). The median age was 51.0 [42.3, 58] years, and 63 patients (73.26%) were men. During a 1-year follow-up, 12/86 patients (13.95%) experienced ipsilateral cerebral ischemic events (Fig. 2) with six patients having the events within 6 months. Of these twelve patients, eight patients (75.0%) had ischemic strokes, and four confirmed TIA. Five patients underwent follow-up MRI scans after the ischemic event, and four showed new infarction in the same territory of the culprit side. The infraction patterns contain two borderzone infarcts and two perforator infarcts.

### The association between baseline characteristics and recurrent events

As shown in Table 1, there was no significant difference in demographics and common laboratory examinations between patients with and without recurrent ischemic events at the baseline. Except for the remodel index (0.76 [0.64, 0.97] vs 0.98 [0.84, 1.09], *p* = 0.043), no significant difference was found in lesion morphologic and compositional features between these two patient cohorts. Patients’ baseline features showed no significance in predicting ischemic events (Supplementary Fig. 1).

**Table 1:** Clinical feature and lesion morphological and compositional features at baseline

	Patients without cerebral ischemic events *n* = 74	Patients with cerebral ischemic events *n* = 12	*p*-value
Age, years	51.5 [45.0, 59.0]	45.0 [32.0, 54.3]	0.118
Male, *n* (%)	53 (71.62%)	10 (83.33%)	0.618
Systolic pressure (mmHg)	130.0 [120.0, 142.8]	140.0 [129.0, 147.3]	0.129
Symptomatic plaque, *n* (%)	61 (82.43%)	10 (83.33%)	0.920
Medical history, *n* (%)			
Hypertension	34 (45.95%)	5 (41.67%)	1.000
Diabetes mellitus	24 (32.43%)	3 (25.00%)	0.858
Smoke	26 (35.14%)	3 (25.00%)	0.719
Previous stroke	12 (16.22%)	3 (25.00%)	0.739
Coronary disease	7 (9.46%)	1 (8.33%)	1.000
TC, mmol/L	3.93 [3.20, 4.98]	4.55 [3.85, 6.35]	0.210
TG, mmol/L	1.27 [0.93, 1.84]	1.58 [0.92, 2.69]	0.533
HDL-C, mmol/L	1.13 [0.90, 1.22]	1.07 [1.00, 1.14]	1.000
LDL-C, mmol/L	2.42 [1.78, 3.18]	2.91 [2.33, 3.90]	0.331
hrMRI feature			
VA_MLA_, mm^2^	13.53 [10.63, 16.18]	11.64 [9.33, 15.55]	0.347
LA_MLA_, mm^2^	1.47 [0.77, 2.37]	1.10 [0.68, 1.68]	0.267
VA_RA_, mm^2^	14.47 [11.99, 16.23]	15.61 [12.65, 18.02]	0.400
LA_RA_, mm^2^	5.86 [5.10, 7.24]	5.72 [4.52, 7.26]	0.722
WT_max_ mm	2.10 [1.85, 2.59]	2.30 [1.80, 2.54]	0.722
Plaque burden	0.89 [0.83, 0.94]	0.90 [0.87, 0.92]	0.443
Remodeling index	0.98 [0.84, 1.09]	0.76 [0.64, 0.97]	0.043*
Eccentric index	0.67 [0.56, 0.74]	0.68 [0.61, 0.71]	0.769
Eccentric plaque (%)	64 (86.49%)	12 (100.00%)	0.385
Positive remodeling, *n* (%)	23 (31.08%)	3 (25.00%)	0.931
IPH, *n* (%)	9 (12.16%)	1 (8.33%)	1.000
Enhancement ratio	2.06 [1.33, 2.91]	2.22 [1.56, 3.07]	0.861
Enhancement rate, *n* (%)			0.530
0	12 (16.22%)	1 (8.33%)	
1	20 (27.03%)	5 (41.67%)	
2	42 (56.76%)	6 (50.00%)	
Degree of stenosis (%)	80.0 [58.0, 89.8]	80.00 [73.0, 87.5]	0.458

*TC* total cholesterol, *TG* triglycerides, *HDL-C* high-density lipoprotein cholesterol, *LDL-C* low-density lipoprotein cholesterol, *VA*_*MLA*_ vessel area at the most stenotic area, *VA*_*RA*_ vessel area at the healthy reference section nearby, *LA*_*MLA*_ minimum luminal area, *LA*_*RA*_ lumen area at the reference site, *WT*_*max*_ maximum wall thickness

* *p* < 0.05

The intra- and inter-observer agreements of hrMRI measurement were good. Details can be found in Supplementary Table S1.

### The association between follow-up hrMRI characteristics and subsequent ischemic events

**Fig. 3:**
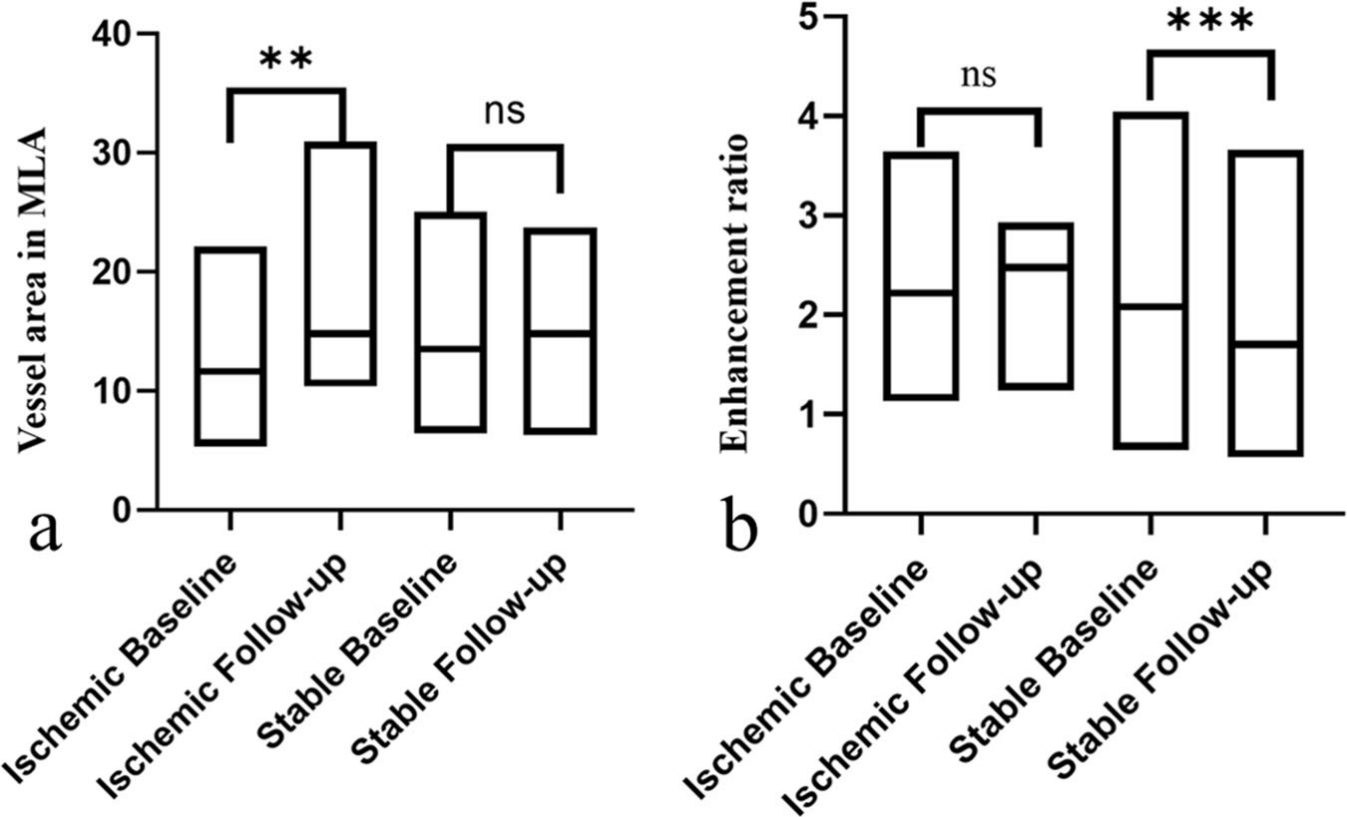
Comparison of vessel area and enhancement ratio between baseline and follow-up in patients with and without cerebral ischemic events. **a** Comparison of vessel area in patients with and without cerebral ischemic events. **p < 0.01 follow-up scans. **b** Comparison of enhancement ratio in patients with and without cerebral ischemic events. ***p < 0.001

**Fig. 4:**
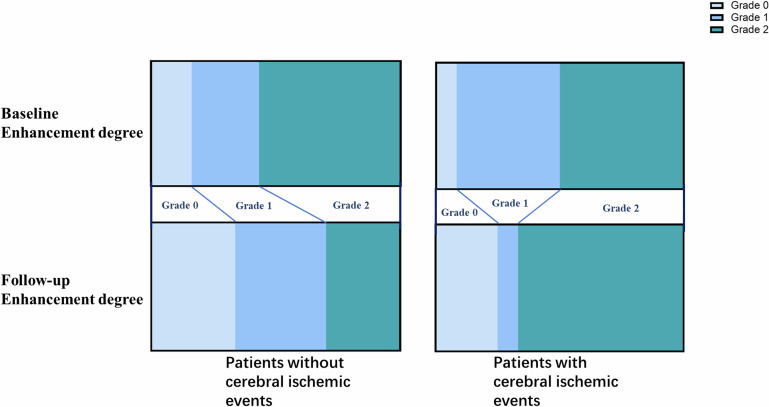
Change of enhancement degree in patients with and without ischemic events. Change of enhancement degree in patients with and without cerebral ischemic events

As shown in Table 2, patients with cerebral ischemic events had higher stenosis rate (80.6 [76.8, 90.1] vs 72.0 [57.4, 84.9], *p* = 0.031), higher plaque burden (0.94 [0.89, 0.97] vs 0.87 [0.79, 0.92], *p* = 0.003) and higher remodel index (1.05 [0.93–1.23] vs 0.95 [0.83, 1.01], *p* = 0.023) than patients without any ischemic event during the follow-up period. The progression of plaque burden (58.3% vs 16.2%, *p* = 0.005) and vessel expansion (83.3% vs 36.5%, *p* = 0.017) were more frequently observed in patients with ischemic events than those without. The change percentage of plaque burden (0.05 [0.01, 0.07] vs −0.04 [−0.09, 0.00], *p* = 0.002) and remodel index (0.26 [−0.01, 1.27] vs −0.21 [−0.14, 0.11], *p* = 0.005) were significantly different between these two cohorts. The vessel area at the most stenotic site was significantly increased (11.64 [9.33, 15.55] vs 14.83 [12.61, 20.57], *p* = 0.003, unit: mm^2^) in patients who suffered from ischemic events during the follow-up, and the enhancement ratio remained unchanged (2.22 [1.56, 3.07] vs 2.47 [1.45, 2.75], *p* = 0.823). However, such a phenomenon was not observed in the group without any ischemic event (vessel area at the most stenotic site: 13.53 [10.63, 16.18] vs 14.78 [11.54, 15.99], *p* = 0.076), and (enhancement ratio: 2.06 [1.33, 2.91] vs 1.73 [1.50, 2.48], *p* < 0.001) was observed (Figs. 3 and 4). More details can be found in the Supplementary Tables S2 and S3.

**Table 2:** Follow-up hrMRI features

	Patients without cerebral ischemic events *n* = 74	Patients with cerebral ischemic events *n* = 12	*p*-value
Months between examinations	3.65 (3.12–4.96)	3.33 (2.80–3.68)	0.088
Follow-up stenosis %	72.02 (57.44–84.90)	80.62 (76.79–90.10)	0.031*
Stenosis change %	−6.50 (−12.00 to 2.67)	−1.92 (−10.00 to 13.81)	0.188
Stenosis change percentage %	−7.31 (−20.00 to 3.31)	−2.24 (−11.73 to 18.35)	0.122
Follow-up eccentric index	0.76 (0.71–0.80)	0.82 (0.75–0.85)	0.080
Eccentric index change	0.07 (−0.04 to 0.20)	0.12 (−0.02 to 0.21)	0.955
Follow-up IPH, *n* (%)	2 (2.70%)	0 (0.00%)	1.000
Follow-up plaque burden	0.87 (0.79–0.92)	0.94 (0.89–0.97)	0.003*
Plaque burden change	−0.03 (−0.07 to 0.00)	0.04 (0.01–0.06)	0.001*
Plaque burden change percentage %	−0.04 (−0.09 to 0.00)	0.05 (0.01–0.07)	0.002*
Plaque progression, *n* (%)	12 (16.22%)	7 (58.33%)	0.005*
Follow-up remodel index	0.95 (0.83–1.01)	1.05 (0.93–1.23)	0.023*
Follow-up positive remodeling, *n* (%)	17 (24.29%)	8 (50.00%)	0.082
Remodel index change	−0.02 (−0.15 to 0.09)	0.20 (−0.03 to 0.38)	0.004*
LA_MLA_ change	0.43 (1.17)	−0.11 (0.76)	0.017*
VA_MLA_ change	0.01 (−0.09 to 0.14)	0.28 (0.15–0.77)	0.004*
Enhancement ratio change, *n* (%)			0.397
Progression	6 (8.12)	3 (25.00%)	
Unaltered	38 (51.35%)	6 (50.00%)	
Regression	30 (41.40%)	3 (25.00%)	
Outerwall change, *n* (%)			0.017*
Shrinkage	22 (29.73%)	1 (8.33%)	
Unaltered	25 (33.78%)	1 (8.33%)	
Expansion	27 (36.48%)	10 (83.33%)	

*LA*_*MLA*_ minimum luminal area, *VA*_*MLA*_ vessel area at the most stenotic site, *IPH* intraplaque hemorrhage

* *p* < 0.05

**Fig. 5:**
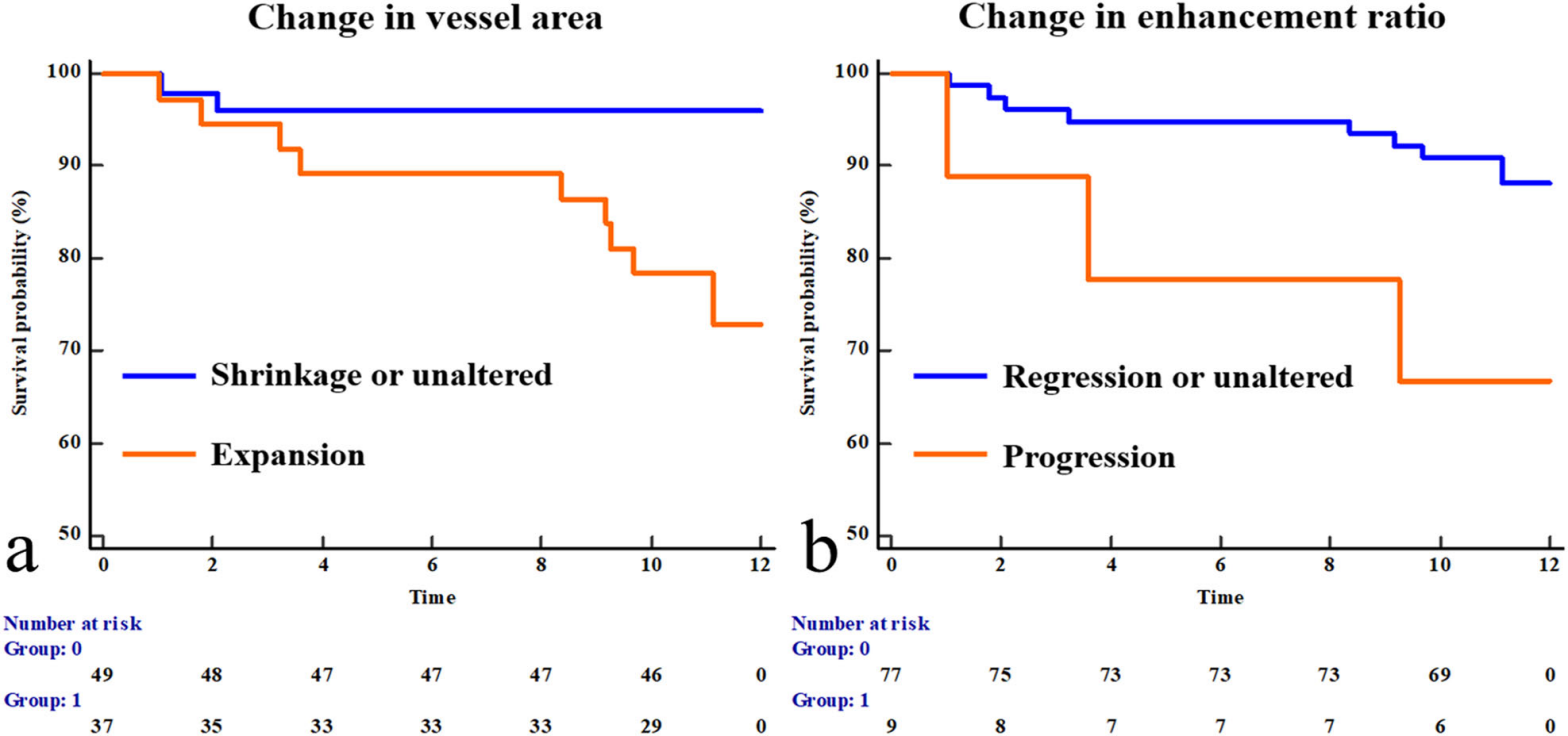
Kaplan–Meier survival curves of patients with MCA stenosis. **a** Kaplan–Meier curve for follow-up ischemic events stratified by change of vessel area during the follow-up (vessel expansion; vessel shrinkage or unaltered, *p* = 0.003 by log-rank test). **b** Kaplan–Meier curves for ischemic events stratified by change of enhancement ratio during the follow-up (enhancement ratio progression, enhancement ratio regression, or unaltered; *p* = 0.045 by log-rank test)

Multivariant Cox regression analysis showed that age (HR = 0.942, 95% CI: 0.903–0.983, *p* = 0.006), progression of plaque burden (HR = 3.818, 95% CI: 1.117–13.051, *p* = 0.033), vessel expansion (HR = 5.173, 95% CI: 1.077–24.838, *p* = 0.040) and enhancement ratio progression (HR = 6.144, 95% CI: 1.480–25.511, *p* = 0.012) were significantly correlated with cerebral ischemic events (Table 3). The C-index of the Cox model was 0.804 (95% CI: 0.658–0.950). Kaplan–Meier survival curves illustrated that vessel expansion (*p* = 0.003) and the enhancement progression (*p* = 0.045) were significantly correlated with a higher ischemic events rate in the 1-year follow-up (Fig. 5).

**Table 3:** Cox regression of risk factors for ipsilateral cerebral ischemic events

	Univariate Cox regression		Multivariate Cox regression	
	HR (95% CI)	*p*-value	HR (95% CI)	*p*-value
Age	0.954 (0.909–1.001)	0.054	0.942 (0.903–0.983)	0.006
Gender (male)	0.536 (0.117–2.445)	0.420		
Hypertension	0.885 (0.281–2.790)	0.835		
Diabetes mellitus	0.738 (0.200–2.725)	0.648		
Previous stroke	1.737 (0.470–6.418)	0.408		
Total cholesterol	1.297 (0.907–1.856)	0.155		
LDL-C	1.216 (0.755–1.960)	0.422		
Baseline stenosis	1.024 (0.984–1.065)	0.251		
Baseline plaque burden	1.050 (0.957–1.152)	0.299		
Progression of plaque burden	5.252 (1.666–16.558)	0.005	3.818 (1.117–13.051)	0.033
Baseline remodel index	0.058 (0.004–0.775)	0.031		
Remodel index change (%)	4.066 (1.850–8.939)	< 0.001		
Vessel expansion	7.205 (1.578–32.898)	0.011	5.173 (1.077–24.838)	0.040
Baseline MLA	0.656 (0.339–1.270)	0.211		
MLA change	0.498 (0.262–0.946)	0.033		
Baseline enhancement degree	1.014 (0.475–2.166)	0.972		
Enhancement ratio change (%)	3.800 (1.185–12.186)	0.025		
Progression of Enhancement ratio	3.324 (0.899–12.293)	0.072	6.144 (1.480–25.511)	0.012

Adjusted for age, gender, hypertension, DM, and previous stroke in multivariate regression analysis

### The association between follow-up hrMRI characteristics and subsequent ischemic events in symptomatic patients

Multivariant Cox regression analysis showed that age (HR = 0.948, 95% CI: 0.907–0.991, *p* = 0.019), vessel expansion (HR = 4.879, 95% CI: 1.002–23.793, *p* = 0.049) and enhancement ratio progression (HR = 4.633, 95% CI: 1.109–19.350, *p* = 0.036) were also significantly correlated with cerebral ischemic events, but progression of plaque burden did not present significance as previous (HR = 3.331, 95% CI: 0.918–12.093, *p* = 0.063) (Table 4). More details of the symptomatic subgroup can be found in the Supplementary Fig. 2.

**Table 4:** Cox regression of risk factors for ipsilateral cerebral ischemic events in symptomatic patients

	Univariate Cox regression		Multivariate Cox regression	
	HR (95% CI)	*p*-value	HR (95% CI)	*p*-value
Age	0.960 (0.914–1.010)	0.113	0.948 (0.907–0.991)	0.019
Gender (male)	0.327 (0.041–2.579)	0.289		
Hypertension	1.089 (0.315–3.764)	0.892		
Diabetes mellitus	1.071 (0.277–4.141)	0.921		
Previous stroke	2.134 (0.552–8.254)	0.272		
Progression of plaque burden	4.416 (1.277–15.267)	0.019	3.331 (0.918–12.093)	0.067
Baseline remodel index	0.020 (0.001–0.333)	0.006		
Remodel index change (%)	22.418 (3.228–155.712)	0.002		
Vessel expansion	6.066 (1.287–28.583)	0.023	4.879 (1.002–23.793)	0.049
Baseline MLA	0.755 (0.388–1.469)	0.408		
MLA change	0.544 (0.276–1.075)	0.080		
Baseline enhancement degree	0.833 (0.357–1.941)	0.671		
Enhancement ratio change (%)	3.989 (1.300–12.233)	0.016		
Progression of enhancement ratio	3.391 (0.876–13.124)	0.077	4.633 (1.109–19.350)	0.036

Adjusted for age, gender, hypertension, DM and previous stroke in multivariate regression analysis

## Discussion

This study showed a disappointing finding that neither traditional patient demographics, laboratory tests, nor lesion morphologic and compositional features at the baseline could identify higher-risk patients with ICAS who would likely suffer from ischemic events. However, it was exciting that the change in lesion morphologic features and inflammation status could be very helpful in predicting higher-risk patients. The key high-risk features included vessel wall expansion at the most stenotic site, increased plaque burden, and remaining high inflammation burden in the follow-up exam.

Previous longitude studies have shown that the progression of plaque burden or stenotic degree may serve as an independent marker for predicting stroke recurrence [21, 22], these conclusions are aligned with this study. The progression of stenosis degree or plaque burden is typically the most straightforward pattern observed during routine imaging follow-ups, indicating a lack of efficacy in medical treatment. Nevertheless, identifying the progression of plaque burden or stenotic degree in patients with initially high rates of stenosis can be challenging. Therefore, it is crucial to identify other significant changes in plaque features to effectively utilize follow-up imaging for patients.

The risky nature of lesion enhancement has been widely observed. It was considered to be closely correlated with local inflammation, increased vascular endothelial permeability, and neovascularization [11], and it has been recognized as a biomarker for culprit plaques in carotid and intracranial arteries [30–32], and for future ischemic events [14, 33–35]. However, some studies have shown that the presence of plaque enhancement during the baseline may not be predictive [36, 37]. The current study found that persistent enhancement was a risk factor. This is in agreement with the observation in the atherosclerotic plaque that the change in plaque enhancement during follow-up was associated with ischemic stroke recurrence [20, 38].

The current study also found that the increase in remodel index and vessel expansion was associated with follow-up cerebral ischemic events. Six patients who suffered from ischemic events in this study progressed from non or negative remodeling to positive remodeling. The status of positive remodeling has been demonstrated to be one of the high-risk features for patient clinical presentations [13, 39]. During the progression of atherosclerosis, the involved arteries undergo morphological changes through various patterns of vascular remodeling to accommodate the development of atherosclerotic lesions [40]. Negative remodeling is considered a stability alteration characterized by relatively stable plaques and gradual reduction in vessel diameter under prolonged low perfusion [29]. However, positive remodeling is widely regarded as a manifestation of plaque instability and is considered one of the vulnerable plaque characteristics in carotid, which is associated with recurrent ischemic cerebrovascular events [41]. Besides, positive remodeling is more common in symptomatic ICAS patients and more relevant to downstream infractions [13, 42].

The conclusion of this study indicates that follow-up hrMRI vessel wall image can provide additional information in patients with ICAS, which may reflect the efficiency of medication treatment. In clinical practice, for patients with ICAS undergoing optimal medical treatment, MRI vessel wall imaging is recommended to be conducted for a re-exam within 3 months to observe the changes in plaques and clarify the drug’s therapeutic effect. If high-risk follow-up hrMRI features like enhancement ratio progression or plaque burden progression were observed, medication adjustment (changing antiplatelet medicine or using PCSK9 inhibitors, etc.) or endovascular treatment [43–45] should be taken into consideration to prevent patients from subsequent ischemic events.

Despite important findings, there are limitations in this study. First, the changes in plaques were only observed and assessed at the imaging level. Our analysis was conducted solely using the baseline laboratory parameters and did not include information during follow-up; second, due to the multicentre and retrospective nature of the study, detailed medication information after discharge varied across centers, such as whether they received intensive statin therapy or dual antiplatelet therapy. Furthermore, ensuring medication adherence among patients from discharge to hospital reexamination or symptom recurrence posed significant challenges.

Lastly, although patients were from three centers, the total number was still small. A prospective, well-designed, and controlled large-scale study is necessary to establish the utility of longitudinal hrMRI in assessing treatment response and preventing stroke in patients with ICAS.

In conclusion, longitude high-resolution magnetic resonance imaging (hrMRI) offers valuable dynamic insights into the evolution of intracranial plaques. Plaque burden progression, vessel expansion, and enhancement ratio progression revealed by longitude hrMRI were associated with future cerebral ischemic events, providing superior predictive capability compared to baseline plaque characteristics.

## Supplementary information

ELECTRONIC SUPPLEMENTARY MATERIAL

## Abbreviations

DWI: Diffusion-weighted imaging
HR: Hazard ratio
hrMRI: High-resolution magnetic resonance vessel wall imaging
ICAS: Intracranial atherosclerosis stenosis
IPH: Intraplaque hemorrhage
MCA: middle cerebral artery
TIA: Transient ischemic attack
TOF: Time-of-flight
