# Supplementary material for: Atherosclerotic plaque evolution predicts cerebral ischemic events in patients with intracranial atherosclerosis: a multicentre longitudinal study using high-resolution MRI
Source: Eur Radiol. 2024 Dec 19;35(6):3238–48. doi: 10.1007/s00330-024-11248-8 (PMC12081491; doi:10.1007/s00330-024-11248-8)
Supplement: Supplementary file 1 — ELECTRONIC SUPPLEMENTARY MATERIAL [file 330_2024_11248_MOESM1_ESM.pdf]

Atherosclerotic plaque evolution predicts cerebral ischemic events in patients with intracranial atherosclerosis: a multicentre longitudinal study using high-resolution MRI

ELECTRONIC SUPPLEMENTARY MATERIAL

1. MR protocol

In Zhongshan hospital, high-resolution, multi-contrast vessel wall magnetic resonance imaging (hrMRI) was performed using a 3.0 Tesla (T) MR scanner (Verio, SIEMENS, Erlangen, Germany) with a dedicated 18-channel phased array head coil. The protocol included, three-dimensional time-of-flight angiography (3D TOF); black blood two-dimensional fast spin-echo (FSE) T_1-weighted imaging (T1WI), echo time (TE)=27 ms; repetition time (TR)=700 ms, number of excitations (NOX)=4, FOV=100×100 mm^2, spacing between slices: 2.4 mm; slice thickness: 2 mm; black blood two-dimensional FSE T_2-weighted imaging (T2WI), TR/TE=3070/56 ms, NOX=3. Field of view (FOV): 130×130 mm^2; spacing between slices: 2.5 mm; slice thickness: 2 mm, matrix: 256×256.

In Jinling hospital, Medical school of Nanjing University, hrMRI was performed using a 3.0T MR scanner (Discovery MR750, GE Healthcare, Waukesha, WI, USA) with a dedicated 32-channel head coil. The protocol included, 3D TOF; T1WI, TR/TE=560/15.9 ms; T2WI, TR/TE=3400/58 ms. The following parameters were used for both T1WI and T2WI sequences, FOV=100×100 mm^2; spacing between slices=2.2 mm; slice thickness=2 mm; matrix=320×256.

In Changhai hospital, high-resolution, multi-contrast vessel wall magnetic resonance imaging (hrMRI) was performed using a 3.0 Tesla (T) MR scanner (Signa HDxT, GE Medical Systems, Waukesha, WI, USA) with a dedicated 8-channel phased array head coil. The protocol included, three-dimensional time-of-flight angiography (3D TOF); black blood two-dimensional fast spin-echo (FSE) T_1-weighted imaging (T1WI), echo time (TE)=16 ms; repetition time (TR)=567 ms; number of excitations (NOX)=2; black blood two-dimensional FSE T_2-weighted imaging (T2WI), TR/TE=2883/50 ms, NOX=3. The following parameters were used for both T1WI and T2WI sequences, field of view (FOV): 100×100 mm^2; spacing between slices: 2.5 mm; slice thickness: 2 mm, matrix: 320×256.

Sup. Fig 1 Forest figure of baseline features in predicting ischemic events

Features	HR(95%CI)	P
Age	0.954(0.909-1.001)	0.054
Gender(Male)	0.536(0.117-2.445)	0.42
Hypertension	0.885(0.281-2.790)	0.835
Diabetes mellitus	0.738(0.200-2.725)	0.648
Smoking	0.632(0.171-2.333)	0.632
Previous stroke	1.737(0.470-6.418)	0.408
Total cholesterol	1.297(0.907-1.856)	0.155
Triglycerides	1.275(0.998-1.628)	0.051
LDL-C	1.216(0.755-1.960)	0.422
Baseline stenosis	1.024(0.984-1.065)	0.251
Baseline plaque burden	1.050(0.957-1.152)	0.299
Baseline remodel index	0.058(0.004-0.775)	0.031
Baseline MLA	0.656(0.339-1.270)	0.211
Baseline Enhancement degree	1.014(0.475-2.166)	0.972

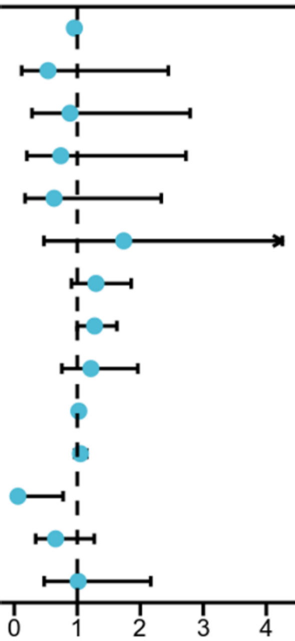

Sup. Tab 1 Intra and interobserver agreement for hrMRI measurement

Measurement	Intra-observer agreement	Inter-observer agreement
VA_{MLA}	0.881 (0.825-0.919)	0.923 (0.738-0.980)
LA_{MLA}	0.804 (0.713-0.866)	0.821 (0.722-0.953)
VA_{RA}	0.859 (0.801-0.902)	0.926 (0.741-0.981)
LA_{RA}	0.772 (0.677-0.841)	0.880 (0.592-0.969)
WT_{max}	0.730 (0.653-0.831)	0.846 (0.518-0.959)
WT_{min}	0.785 (0.694-0.849)	0.890 (0.638-0.971)
T1-CE signal	0.791 (0.705-0.853)	0.966 (0.876-0.991)
T1-CE signal of reference	0.731 (0.569-0.828)	0.893 (0.632-0.972)
T1 signal	0.830 (0.749-0.885)	0.852 (0.517-0.961)
T1signal of reference	0.871 (0.806-0.914)	0.929 (0.752-0.982)

VA_{MLA}, vessel area at the most stenotic site; VA_{RA}, vessel area at the healthy reference section nearby; LA_{MLA}, minimum luminal area; LA_{RA}, lumen area at the reference site; WT_{max}, maximum wall thickness.

Sup. Tab 2 Paired T-test in patients without ischemic events

	Baseline (n=74)	Follow-up (n=74)	*p* value
VA_{MLA} (mm^2)	13.53 (10.63-16.18)	14.78(11.54-15.99)	0.076
VA_{RA} (mm^2)	14.47 (11.99-16.23)	14.93(12.31-19.34)	0.002
LA_{MLA} (mm^2)	1.47 (0.77-2.37)	2.00(1.03-2.77)	<0.001
LA_{RA} (mm^2)	5.86 (5.10-7.24)	6.78(5.42-8.21)	0.023
WT_{max} (mm)	2.10 (1.85-2.59)	2.21(1.74-2.60)	0.936
Enhancement degree (%)			
0	12 (16.22%)	25(33.78%)	<0.001
1	20 (27.03%)	27(36.49%)	
2	42 (56.76%)	22(29.73%)	
Enhancement ratio	2.06(1.33-2.91)	1.73(1.50-2.48)	<0.001
Plaque burden	0.89 (0.83-0.94)	0.92(0.87-0.94)	0.001
Remodeling index	0.98 (0.84-1.09)	1.00(0.80-1.12)	0.542
Eccentric index	0.67 (0.56-0.74)	0.78(0.75-0.82)	<0.001

VA_{MLA}, vessel area at the most stenotic site; VA_{RA}, vessel area at the healthy reference section nearby; LA_{MLA}, minimum luminal area; LA_{RA}, lumen area at the reference site; WT_{max}, maximum wall thickness.

Sup. Tab 3 Paired T-test in patients with ischemic events

	Baseline (n=12)	Follow-up (n=12)	*p* value
VA_{MLA} (mm^2)	11.64 (9.33-15.55)	14.83(12.61-20.57)	0.003
VA_{RA} (mm^2)	15.61 (12.65-18.02)	14.22(11.41-19.86)	0.960
LA_{MLA} (mm^2)	1.10 (0.68-1.68)	1.16(0.58-1.55)	0.428
LA_{RA} (mm^2)	5.72 (4.52-7.26)	7.34(4.06-8.25)	0.308
WT_{max} (mm)	2.30 (1.80-2.54)	2.64(2.29-3.20)	0.032
Enhancement degree (%)			
0	1 (8.33%)	3(25.00%)	1.000
1	5 (41.67%)	1(8.33%)	
2	6 (50.00%)	8(66.67%)	
Enhancement ratio	2.22(1.56-3.07)	2.47(1.45-2.74)	0.823
Plaque burden	0.90 (0.87-0.92)	0.94(0.88-0.97)	0.127
Remodeling index	0.76 (0.64-0.97)	1.05(0.88-1.36)	0.022
Eccentric index	0.68 (0.61-0.71)	0.85(0.82-0.88)	0.047

VA_{MLA}, vessel area at the most stenotic site; VA_{RA}, vessel area at the healthy reference section nearby; LA_{MLA}, minimum luminal area; LA_{RA}, lumen area at the reference site; WT_{max}, maximum wall thickness.

Sup Fig 2 Forest figure of features in predicting ischemic events in symptomatic patients

Characteristic	HR(95%CI)	P value
Gender (Male)	0.327(0.041-2.579)	0.289
Hypertension	1.089(0.315-3.764)	0.892
Diabetes mellitus	1.071(0.277-4.141)	0.921
Previous stroke	2.134(0.552-8.254)	0.272
Progression of plaque burden	4.416(1.277-15.267)	0.019
Baseline remodel index	0.020(0.001-0.333)	0.006
Vessel expansion	6.066(1.287-28.583)	0.023
Baseline MLA	0.755(0.388-1.469)	0.408
MLA change	0.544(0.276-1.075)	0.08
Baseline Enhancement degree	0.833(0.357-1.941)	0.671
Enhancement ratio change (%)	3.989(1.300-12.233)	0.016
Progression of Enhancement ratio	3.391(0.876-13.124)	0.077

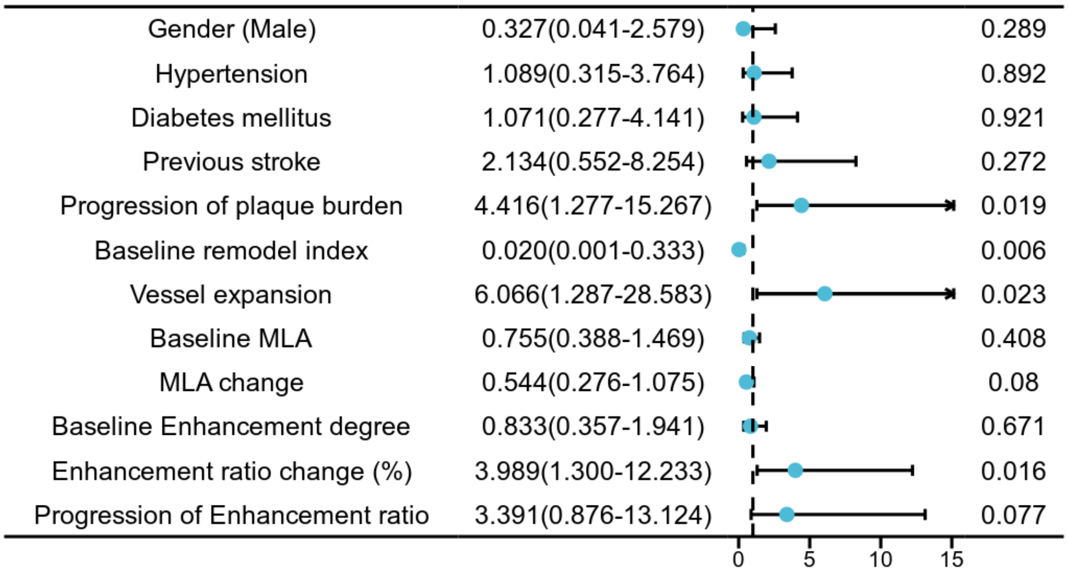

Sup Fig 3 Image analysis procedure

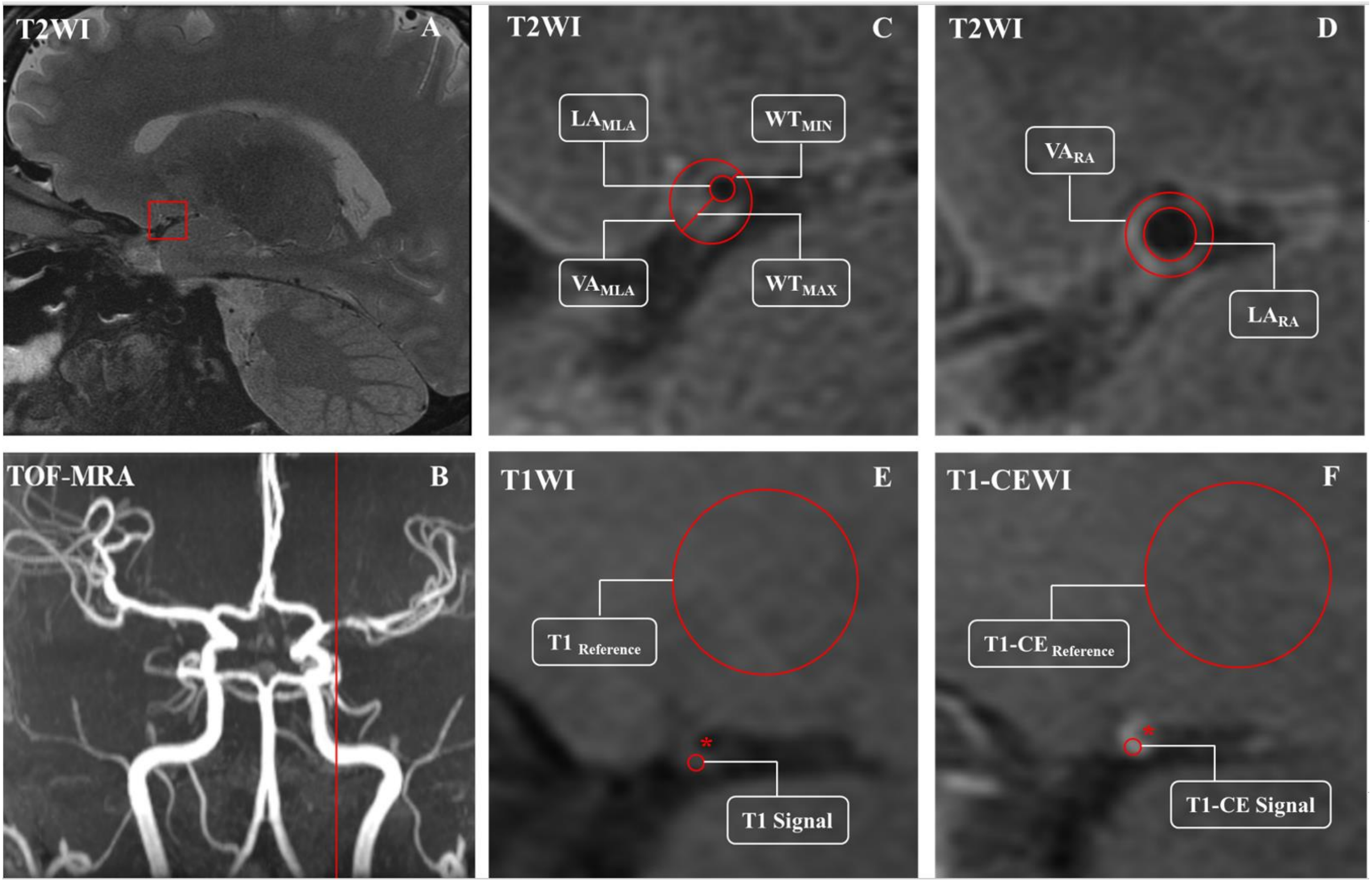

Image analysis procedure of vessel wall image. Firstly, in the black blood 2D-T2 sequence (A), the most stenotic and reference planes were clarified concerning the TOF-MRA sequence (B). We draw the lumen and vessel outer wall with a closed polygon tool in the most stenotic (C) and reference (D) planes and get their areas accordingly. The maximum and minimum plaque thickness passing through the center of the lumen is measured using a linear measurement tool (C). Finally, the signal intensity of the vessel wall and adjacent reference sites is measured in T1 (E) and T1-CE (F) sequences. Based on the above measurement parameters, the plaque features, such as plaque burden, eccentricity index, remodeling index, enhancement index, etc., are then calculated according to the formula. *Lumen
